# Resolvin E1 in Follicular Fluid Acts as a Potential Biomarker and Improves Oocyte Developmental Competence by Optimizing Cumulus Cells

**DOI:** 10.3389/fendo.2020.00210

**Published:** 2020-04-16

**Authors:** Yijing Zhang, Zhongyi Zhu, He Li, Mingjiang Zhu, Xiandong Peng, Aijie Xin, Ronggui Qu, Wen He, Jing Fu, Xiaoxi Sun

**Affiliations:** ^1^Obstetrics and Gynecology Hospital of Fudan University, Shanghai, China; ^2^Shanghai JIAI Genetics & IVF Institute, Shanghai, China; ^3^Key Laboratory of Female Reproductive Endocrine Related Diseases of Obstetrics and Gynecology Hospital of Fudan University, Shanghai, China; ^4^CAS Key Laboratory of Nutrition, Metabolism and Food Safety, Shanghai Institute of Nutrition and Health, Shanghai Institutes for Biological Sciences (SIBS), Chinese Academy of Sciences (CAS), Shanghai, China

**Keywords:** metabolomics, resolvin E1, follicular fluid, cumulus cells, oocyte quality

## Abstract

Metabolic profile of follicular fluid (FF) has been investigated to look for biomarkers for oocyte quality. Resolvin E1 (RvE1), a potent pro-resolving mediator, was reported to have protective action in cell function. The study aimed to examine the predictive value of RvE1 for oocyte quality and to explore the cellular mechanism of RvE1 in improving oocyte competence. Metabolic profiles of 80 FF samples showed a higher level of RvE1 in group A (blastocysts scored ≥ B3BC and B3CB according to Gardner's blastocyst scoring system, *N* = 36) than that of group B (blastocysts scored < B3BC and B3CB, *N* = 44, *P* = 0.0018). The receiver operating characteristic (ROC) curve analysis showed that RvE1 level in FF below 8.96 pg/ml (AUC:0.75; 95%CI: 0.64–0.86; *P* = 0.00012) could predict poor oocyte quality with specificity of 97.22%, suggesting RvE1 as a potential biomarker to exclude inferior oocytes. Besides, the level of RvE1 was found to be significantly lower in FF than in serum (57.49 to 17.62 pg/ml; P=.0037) and was gradually accumulated in the culture medium of cumulus cells (CCs) during cell culture, which indicated that RvE1 came from both blood exudates and local secretion. The in vitro experiment revealed thecellular mechanism of RvE1 in improvingoocyte qualityby decreasing the cumulus cellapoptotic rate and increasing cell viability and proliferation. It is the first time thatthe role of RvE1 in reproduction is explored. In conclusion, RvE1 is valuable as a potential exclusive biomarker for oocyte selection andplays a role in improving oocyte quality.

## Introduction

With an increasing number of infertile couples worldwide, *in vitro* fertilization (IVF) has become the most effective treatment for infertility in both developed and developing countries. Nevertheless, the success rate of IVF is still below 40% even in the most advanced areas (1, 2). In an attempt to optimize the IVF outcome, recent researches have focused on the identification of non-invasive biomarkers to aid the selection of oocytes and embryos with superior quality and viability (3, 4).

Follicular fluid (FF), the relatively independent microenvironment of oocytes, is produced by the effusion of blood plasma and secretion of theca cells, granulosa cells as well as oocytes (5). FF contains a series of metabolites critical for oocyte maturation and thus its variation in composition, to a certain extent, reflects oocyte developmental competence and embryo viability (6, 7). Therefore, the investigation into the metabolic profile of FF might provide potential biomarkers for oocyte and embryo quality which could be applied as a supplemental assessment method in assisted reproductive technology (ART).

As a systematic analyzing tool for profiling metabolites, metabolomics was characterized for high resolution, sensitivity, and throughput in detecting a large population of molecules in various biological samples (8–10). In the last decade, the advance in metabolomic techniques allowed the identification of numerous individual chemicals in a small volume of biofluid. Basic technologies include mass spectroscopy (LC-MS/MS), gas chromatography-mass spectrometry (GC-MS/MS), liquid chromatography–capillary electrophoresis–mass spectroscopy (CE-MS/MS), and nuclear magnetic resonance spectroscopy (NMRS). Liquid chromatography-electrospray ionization tandem mass spectrometry (LC/ESI-MS/MS), a special LC-MS/MS, facilitates ionization of the majority of targeted subjects and enjoys a wide range of application (11–13). Recently, metabolomics has been applied to lipidomic profiles in the IVF-related fluids such as the culture media of oocytes and embryos and FF aspirated during the oocyte retrieval, searching for possible physiological indicators. However, as to our knowledge, no study has successfully screened out resolvin E1 (RvE1) or demonstrated its correlation with oocyte competence or embryo viability (14, 15).

RvE1, namely 5,12,18R-trihydroxy-eicosapentaenoic acid, is a potent mediator for anti-inflammation and pro-resolving (16). In humans, there are two routes of RvE1 biosynthesis, aspirin-dependent and aspirin-independent. Via cell-cell interactions, RvE1 is formed in inflammatory exudates with aspirin-acetylated cyclooxygenase (COX)-2 and lipoxygenase. Endogenously, it can also be produced via cytochrome P450 conversion of its precursor eicosapentaenoic acid which is derived from omega-3 fatty acid (17, 18). RvE1 exerts bioactivities of anti-inflammation, pro-resolving, and tissue protection by binding to its protein-coupled receptor, ChemR23, on various cells, including monocytes, macrophages, dendritic cells, NK cells and endothelial cells (19). Specifically, RvE1 induces an anti-inflammatory effect, both acutely and chronically, by blocking the production of proinflammatory cytokines (20), inhibiting neutrophil transendothelial migration (17) and enhancing phagocytosis of microbial particles and apoptotic neutrophils (21). Apart from inflammation mediation, RvE1 also functions protectively by accelerating the restoration of endothelium function (22), alleviating tissue damage (23) and promoting bone preservation (24). Furthermore, recent researches have verified the beneficial effects of RvE1 on atherogenesis (25), asthma (26) and Alzheimer's disease (27).

Cumulus cells (CCs) together with the enclosed oocyte function as integrity called cumulus-oocyte complex (COC). The liquid microenvironment of FF and the proximity of these cells to each other allows the bidirectional communication between oocytes and surrounding CCs. The cross-talk between CCs and oocytes is paramount for energy balance and meiosis resumption within COCs. CCs nurture oocyte growth and support cytoplasmic maturation by supplying substrate for ATP production, exporting cyclic guanosine monophosphate (cGMP) and transporting RNAs to regulate the oocytes' gene transcription (28–30). It was found that human denuded oocytes cultured with CCs were found to improve oocyte maturation and subsequent preimplantation development (31). Furthermore, the interactions between the oocyte and the CCs were revealed to significantly influence oocyte maturation rates and were found to be essential for generating embryos with high developmental competence (32). The association between CC apoptotic gene expression and embryo morphogenetics and cleavage pattern was also demonstrated (33). Therefore, CCs play an essential role in oocyte developmental competence and embryo viability in their early stage.

In this study, we conducted a metabolomic analysis of 80 FF samples from IVF patients and correlated individual FF composition to subsequent outcomes of oocytes and embryos, exploring the correlation between RvE1 concentration in FF and the outcomes of corresponding oocytes. Although recent studies have examined the function and mechanism of RvE1 in several systems including respiratory, digestive, motor and nervous systems, the effect of RvE1 on infertility has yet to be fully elucidated. Thus, the aims of this study are: (i) To examine the prediction value of RvE1 as a biomarker for oocyte quality and embryo viability. (ii) To explore the cellular mechanism of RvE1 in improving oocyte developmental competence. (iii) To investigate possible sources of RvE1.

## Materials and Methods

### Ethical Approval

The research protocols were approved by Shanghai Ji Ai Genetics & IVF Institute Ethics Committee (JIAI E2016-08) and written informed consent was obtained prior to sample collection by all participants.

### Patients' Characteristics

This study included a total of 141 IVF patients undergoing controlled ovarian stimulation of GnRH antagonist (GnRH-ant) procedures at the Shanghai Ji Ai Genetics & IVF Institute of Obstetrics and Gynecology Hospital of Fudan University, China. Considering different purposes, the two batches of samples were collected with different inclusion criteria. The first 80 samples of FF were obtained from first follicles in one side or both sides of ovaries of 65 patients with subfertility factors including tubal factor, polycystic ovary syndrome (PCOS), endometriosis, male factor, genetic disease, and other unidentified etiological factors and only the first or the second cycle was adopted. In the next group, 76 subjects were enrolled and similarly, only the first or the second cycle was included. To eliminate the interference of abnormal metabolic level, PCOS, endometriosis and other metabolism-related diseases were excluded. In this part of the research, indications for IVF were strictly limited to tubal factor, male factor, idiopathic and aging. The hormonal status baselines of patients from two groups were shown in Table 1 and Supplementary Table 1, respectively.

**Table 1:** Clinical characteristics of subjects undergoing follicular fluid metabolic analysis by liquid chromatography electrospray ionization tandem mass spectrometry (LC/ESI-MS/MS).

**Clinical parameter**	**Group A (*N* = 36)**	**Group B (*N* = 44)**	***P*-values**
Age (years)	29.7 ± 3.4	29.6 ± 3.4	0.921^a^
BMI (kg/m^2^)	21.4 ± 2.5	22.8 ± 4.0	0.060^a^
E2 baseline (pg/ml)	37.1 ± 10.3	38.7 ± 17.7	0.644^a^
P4 baseline (ng/ml)	0.6 ± 0.3	0.5 ± 0.3	0.624^a^
FSH baseline (mIU/ml)	7.0 ± 1.9	7.8 ± 2.8	0.157^a^
LH baseline (mIU/ml)	4.8 ± 1.9	6.5 ± 9.6	0.285^a^
Oocyte score	2.9 ± 0.5	2.9 ± 0.4	0.477^a^
Fertilization	2.0 ± 0.4	2.0 ± 0.2	0.323^a^
2pn	34 (94.4%)	43 (97.7%)	0.177^c^
1pn	1 (2.8%)	1 (2.3%)	1.000^b^
0pn	1 (2.8%)	0 (0%)	0.450^d^
Peter score on Day 3	1.9 ± 0.5	2.3 ± 0.7	0.003^a^
Embryo on Day 3			0.0031^d^
Top quality embryos	33 (91.7%)	32 (72.7%)	
(grade 1–2)			
Poor quality embryos	3 (8.3%)	12 (27.3%)	
(grade 3–4)			
Presence of blastomeres	8.3 ± 1.3	6.3 ± 3.0	0.0001^a^
Embryos with <6 cells	0 (0%)	18 (40.9%)	<0.0001^d^
Embryos with 6–8 cells	26 (72.2%)	15 (34.1%)	0.001^d^
Embryos with >8 cells	10 (27.8%)	11 (25.0%)	0.779^d^
Subfertility			0.062^d^
Primary	18 (50.0%)	31 (70.5%)	
Secondary	18 (50.0%)	13 (29.5%)	
Etiology of infertility			
Tubal factor	18 (50.0%)	27 (61.4%)	0.308^d^
Endometriosis	2 (5.6%)	2 (4.5%)	0.309^c^
PCOS	0 (0%)	1 (2.3%)	1.000^b^
Male factor	21 (58.3%)	30 (68.2%)	0.362^d^
Genetic disease	9 (25.0%)	6 (13.6%)	0.195^d^
Idiopathic	4 (11.1%)	5 (11.4%)	0.749^c^
PGS	10 (27.8%)	6 (13.6%)	0.115^d^

*Data are mean ± SD. Group A: subjects with high-quality blastocysts (blastocyst scored ≥ B3BC and B3CB according to Gardner's blastocyst scoring system); Group B: subjects with low-quality blastocysts (blastocyst scored < B3BC and B3CB). P-values: a, unpaired t-test with two tails; b, Fisher's exact test; c, Yates 'continuity corrected chi-square test; d, Chi-squre test. BMI, body mass index; E2, estradiol; P4, progesterone; LH, luteinizing hormone; FSH, follicle stimulating hormone; PCOS, polycystic ovary syndrome; pn: pronuclear; Day 3 embryo quality was graded according to modified Peter cleavage stage embryos scoring system with morphological criteria of number of blastomeres, blastomere regularity, granule occurrence and fragmentation rate; PGS, preimplantation screening*.

### IVF Procedure and Sample Collection

Controlled ovarian stimulation was performed using GnRH-ant (Cetrotide; Merck- Serono, Geneva, Switzerland) and recombinant FSH (Gonal F, Merck-Serono, Geneva, Switzerland). To trigger ovulation, 10,000 IU HCG (Profasi; Merck-Serono, Geneva, Switzerland) was administered when the dominant follicle grew up to over 18 mm and/or serum estradiol levels exceeded 0.8 nM. Oocytes were retrieved 36 h later for IVF or ICSI and embryos were cultured for 6 days at 37°C with 6% CO_2_ in the air. For the 80 samples, only the FF from the first follicle was collected and the outcome of corresponding oocytes and embryos were closely tracked. To compare RvE1 content in serum and FF, we collected and pooled FF samples individually without flushing or anesthesia and selected 76 samples without blood contamination.

### Liquid Chromatography-Electrospray Ionization Tandem Mass Spectrometry (LC/ESI-MS/MS)

Samples of FF and serum were centrifuged at 300 g for 5 min and the supernatant was reserved at −80°C before transporting to a laboratory for quantification with dry-ice. Metabolites of arachidonic acid (AA) and other polyunsaturated fatty acids were analyzed by LC/ESI-MS/MS for targeted metabolomic assessment. A mixture of deuterated internal standards was added to 400 μl FF and 1 N HCl was used to adjust the pH to 3.0. We conducted liquid-liquid extraction twice using hexane: methyl t-butyl ether (50:50, v/v) with a gradient of mobile phase A (water: acetonitrile: formic acid 63:37:0.02, v/v/v) and mobile phase B (acetonitrile: isopropanol 50:50, v/v), FF samples were separated on a Phenomenex Kinetix C18 column (3 μm, 100 × 2.1 mm; Phenomenex, Torrance, CA) using Thermo Accela Ultra Performance Liquid Chromatography (UPLC) system (Thermo Fisher Scientific Life Sciences, Massachusetts, USA). TSQ Vantage triple-quadrupole mass spectrometer (Thermo Fisher Scientific Life Sciences, Massachusetts, USA) was utilized to analyze the mass spectrometry of FF in a negative ion mode and X caliber software (version 2.0, Thermo Fisher Scientific Life Sciences, Massachusetts, USA) was applied to analyze data. According to our previously published literature (34), a systematic metabolomic analysis was performed to detect RvE1 in FF. Briefly, after injection of FF onto a Phenomenex C18 column (2.6 μm, 100 × 2.1 mm; Phenomenex, Torrance, CA) at a flow rate of 400 μm per minute, samples were analyzed by AB Sciex 5500 QTrap hybrid quadrupole linear ion trap mass spectrometer in negative ion mode. Gradient initiated with 100% to 92% A (H_2_O/ACN/FA, 63/37/0.02, by vol) in the first 6 min and was held at 45% A from 6.5 to 10 min. At 13 min, it increased to 100% B (ACN/ IPA, 50/50, by vol) while from 14 to 14.5 min, returned to 100% A. Serum RvE1 was quantified by a targeted metabolomics method with modification to a previously published LC-MS/MS approach (11, 35). In brief, a mixture of 20 deuterated internal standards was added to 200 μl serum and 1 N HCl was used to adjust the pH to 3.0. Liquid-liquid extraction of the mixture was conducted twice using hexane: methyl t-butyl ether (50:50, v/v). After centrifugation at 1,258 g for 8 min, the upper layer was transferred to a new glass tube and the combined organic phase was evaporated under a gentle stream of nitrogen. The residue was reconstituted in 50 μl mobile phase A and stored at −80°C. Serum samples were injected onto a Phenomenex Kinetix C18 column (3 μm, 100 × 2.1 mm) at a flow rate of 0.4 ml per minute by the Thermo Accela UPLC system. RvE1 was then extracted by a gradient of mobile phase A (water: acetonitrile: formic acid 63:37:0.02, v/v/v) and mobile phase B (acetonitrile: isopropanol 50:50, v/v). Mobile phase A, starting from 100% and decreased to 92% in the first 6 min, dropped to 45% A within 30 s and was held for 3.5 min. Afterward, it decreased to 0% in 3 min and was held for another 1 min before returning to 100% in 0.5 and holding for 1.5 min. TSQ Vantage triple-quadrupole mass spectrometer (San Jose, CA, USA) was utilized with a stainless-steel capillary (100 μm inner diameter) to fit electrospray ionization source and mass spectrometry was operated in a negative-ion mode using multiple-reaction monitoring. Data were acquired and analyzed by X caliber software.

### Cell Collection and Culture

CCs denuded from oocytes of ten to twenty patients were mixed together to reach an adequate amount for follow-up study and were transported immediately to the laboratory at room temperature. Cells were gently pipetted and centrifuged at 300 g for 5 min. The supernatant was discarded and the precipitate was resuspended in 500 μl hyaluronidase for 5 min. After addition of 2 ml Dulbecco's Modified Eagle Medium/Nutrient Mixture F-12 (DMEM/F-12; Thermo Fisher Scientific Life Sciences, Massachusetts, USA) with 10% fetus bovine serum (FBS; Gibco, Grand Island, NE) and 1% penicillin-streptomycin (PS; Gibco, Grand Island, NE) solution, CCs were centrifuged at 300 g for 5 min and resuspended in 20 ml DMEM/F-12 medium with 10% FBS and 1% PS and plated for later use. As a positive control of ChemR23, THP-1 cell line (American Type Culture Collection, ATCC, Manassas, VA, USA) was thawed quickly in the water bath at 37°C and cultured in 1640 medium (Gibco, Grand Island, NE) with 10% FBS and 1% PS for further test. CCs and THP-1 were adherently cultured in T25 flask (Corning, NY, USA) at 37°C under 5% CO_2_ in the air before use.

### Western Blot

After being washed with cold PBS (Gibco, Grand Island, NE), cells were lysed in 200 μl RIPA lysis buffer (Beyotime Biotechnology, Shanghai, CN) containing 1% phenylmethanesulfonyl fluoride (PMSF; New Cell & Molecular Biotech, Suzhou, CN) and were vortexed briefly and centrifuged at 20,000 g for 10 min, 4°C. The supernatant was collected and protein concentration was determined using a Bradford protein assay kit (Bio-Rad, California, USA). Equal quantities of proteins (30 μg per well) were subjected to SDS-PAGE gels (10%) and transferred to polyvinylidene difluoride (PVDF; Millipore, Bedford, MA, USA) membranes. PVDF membranes were incubated in Tris-Buffered Saline-Tween (TBS-T, 10 mM Tris-HCl, 100 mM NaCl, and 0.5% Tween-20) with 5% non-fat milk for 1 h and then incubated with diluted antibodies to ChemR23 (1:1,000, ab64881, Abcam, Cambridge, UK) and β-actin (1:1,000, AA128, Beyotime Biotechnology, Shanghai, CN) at 4°C overnight. After washing, membranes were blocked with 5% non-fat milk in TBS-T and probed with peroxidase-conjugated secondary antibody to rabbit or mouse (BL003A and BL001A, Biosharp, Hefei, CN), respectively (1:2,000). The immunostainings were detected with an ECL kit (Millipore Sigma, Billerica, USA) and chemiluminescence captured by Gene Tools software (Syngene, Cambridgeshire, UK).

### Immunofluorescence Detection

CCs were seeded into 4-well plates (Nunc, Thermo Fisher Scientific Life Sciences, Massachusetts, USA) at a density of 1.2 × 10^5^ cells per well and oocytes were cultured in 4-well plates before analysis. Both CCs and oocytes were washed three times with cold PBS (Sigma, Billerica, USA) and fixed in 2% PFA for 20 min. After washing three times with Triton X-100 containing Immunol Staining Wash Buffer (Beyotime Biotechnology, CN), fixed samples were stained for 90 min at room temperature by 6 μl SYBR-labeled mouse-anti-human ChemR23 antibody (PA550932, Thermo Fisher Scientific Life Sciences, Massachusetts, USA) diluted with 300 μl Immnol Fluorence Staining Secondary Antibody Dilution Buffer (Beyotime Biotechnology, CN). Cells were washed again with Immunol Staining Wash Buffer for three times. The anti-fluorescence-quenching agent with DAPI (6-diamidino-2-phenylindole; DAPI Fluoromount-G TM; Yeasen Biotechnology, CN) was used to label nuclei. Images of CCs were captured with a fluorescence microscope (NE900, Nexcope, USA) in plates. For oocytes in plates, cells were gently shifted to the glass slide, slipcovered and scanned by the same microscope.

### Cell Viability Assays

CCs were seeded into 96-well plates at a density of 2 × 10^4^ cells per well and were cultured for 24 h. Cells were incubated in culture medium with RvE1 (Cayman Chemicals, Ann Arbor, MI, USA) at a series of concentrations of 1, 10, and 100 nM for a different duration. An equal volume of ethanol was set as control. Cell Counting Kit-8 ({WST-8 [2-(2-methoxy-4-nitrophenyl) −3-(4-nitrophenyl) −5-(2,4-disulfophenyl) −2H- tetrazolium, monosodium salt]} thyou, CCK8; Dojindo Laboratories, Kumamoto, Japan) solution was added to each well and incubated at 37°C for 4 h. Optical density (OD) values were measured at 450 nm using a Microplate Reader (Bio Tek, Vermont, USA).

### Cell Proliferation Assays

Cell proliferation was assessed by the RTCA S16 System (ACEA Biosciences, San Diego, CA). CCs were resuspended and loaded into xCELLigence cell culture E16-Plate (ACEA Biosciences, San Diego, CA) at a density of 2 × 10^4^ cells per well for 24 h. By Label-free Real-time Cellular Analysis System (RTCA; Roche, Penzberg, Germany), cell growth index was normalized at the time point of treatment and detected automatically for the following days.

### Flow Cytometry for Cell Apoptosis and Cell Cycle

CCs were plated in 24-well plates at 2 × 10^5^ cells per well for 24 h and were incubated in culture medium with 10 nM RvE1 for an additional 8 h. Washed with PBS, cells were prepared for apoptosis with the PE Annexin V Apoptosis Detection Kit I (BD Biosciences, New Jersey, USA). As for cell cycle assays, we fixed cells with 70% ethanol and stained them with PI/RNase Staining Buffer (BD Biosciences, New Jersey, USA) after incubation at 4°C overnight. FAC Station (FV500, Beckman Coulter, Brea, USA) was utilized to detect cell apoptosis ratio and cell cycle profile, and Flow Jo software (version 10.4, Flow Jo, Oregon, USA) was used to analyze data.

### Statistical Analysis

All data were analyzed by Prism software (version 6.0c, GraphPad, San Diego, US) and presented as means ± standard deviation (SD) or standard error of the mean (SEM) of at least three independent replicates in the figure legends (details were described in legends). Student's *t*-test with two tails was used to compare variables between two groups and multiple unpaired *t*-tests with two tails using Holm-Sidak correction were applied to statistically assess differences among more than two groups; Chi-squre test, Yates 'continuity corrected chi-square test and Fisher's exact test were applied to compare rates between two groups (details were given in each legend). For all comparisons, *P* < 0.05 was regarded as statistically significant and *P*-values were given in full.

## Results

### The Higher Level of RvE1 in FF Is Related to Oocytes Which Are Able to Form Blastocysts of High Quality

**Figure 1:**
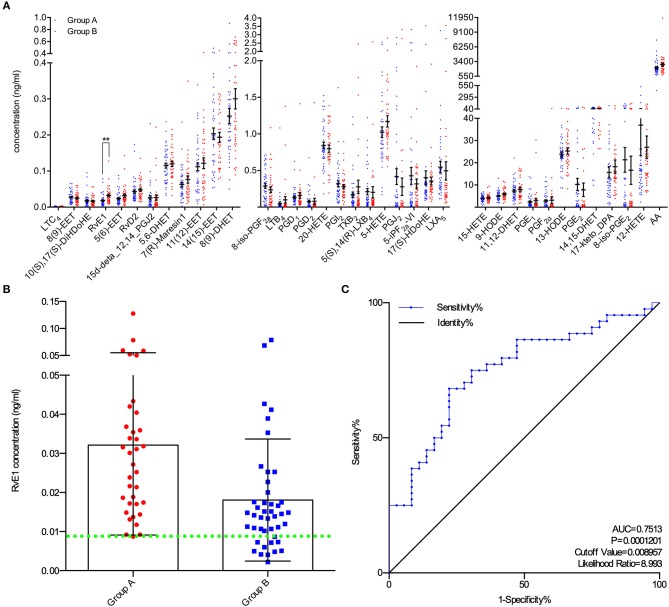
Higher level of RvE1 in follicular fluid (FF) related to oocytes which were able to form blastocysts of high quality. **(A)** Liquid chromatography electrospray ionization tandem mass spectrometry (LC/ESI-MS/MS) was applied to quantitatively analyze metabolite profiles in the FF of oocytes which were able (*N* = 36) or unable (*N* = 44) to form high-quality blastocysts. Data are presented as mean ± SEM; ^**^p = 0.0018, *t*-test with two tails, independent biological replicates number as shown after N. LTC_4_, Leukotriene C_4_; 8(9)-EET, 8,9-epoxyeicosatrienoic acid; 10(S),17(S)-DiHDoHE, 10(S),17(S)-dihydroxy-4Z,7Z,11E,13Z,15E,19Z-docosahexaenoic acid; RvE1, Resolvin E1, 5S,12R,18R-trihydroxy-6Z,8E,10E,14Z,16E-eicosapentaenoic acid; 5(6)-EET, 5,6-epoxy-8,11,14-eicosatrienoic acid; RvD2, Resolvin D2, (4Z,7R,8E,10Z,12E,14E,17S,19Z)-7,16,17-trihydroxy-4,8,10,12,14, 19-docosahexaenoic acid; 15d-deta_12,14_PGJ2, 15-deoxy-Δ^12,14^-Prostaglandin J_2_, 15-deoxyprostaglandin J2; 5,6-DHET, 5,6-dihydroxy-8Z,11Z,14Z-eicosatrienoic acid; 7(R)-Maresin1, 7R,14S-dihydroxy-4Z,8E,10E,12Z,16Z,19Z-docosahexaenoic acid; 11(12)-EET, 11, (12)-epoxy-5Z,8Z,14Z-eicosatrienoic acid; 14(15)-EET, 14, (15)-epoxy-5Z,8Z,11Z-eicosatrienoic acid; 8(9)-DHET, 8,9-dihydroxy-5Z,11Z,14Z-eicosatrienoic acid; 8-iso-PGF_2a_, 8-isoprostaglandin F_2α_, 9α,11α,15S-trihydroxy-(8β)-prosta-5Z,13E-dien-1-oic acid; LTB_4_, Leukotriene B_4_, 5S,12R-dihydroxy-6Z,8E,10E,14Z-eicosatetraenoic acid; PGD_1_, Prostaglandins D_1_, 9α,15S-dihydroxy-11-oxo-prost-13E-en-1-oic acid; PGD_2_, Prostaglandins D_2_, 9α,15S-dihydroxy-11-oxo-prosta-5Z,13E-dien-1-oic acid; 20-HETE, 20-hydroxy-5Z,8Z,11Z,14Z-eicosatetraenoic acid; PGI_2_, Prostaglandin I_2_, 6,9α-epoxy-11α,15S-dihydroxy-prosta-5Z,13E-dien-1-oic acid; TXB_2_, thromboxane B_2_ 9α,11,15S-trihydroxythromba-5Z,13E-dien-1-oic acid; 5(S),14(R)-LXB_4_, 5(S),14(R)-lipoxin B4; 5-HETE, 5-hydroxy-6E,8Z,11Z,14Z-eicosatetraenoic acid; PGJ_2_, 11-oxo-15S-hydroxy-prosta-5Z,9,13E-trien-1-oic acid; 5-iPF_2a_-VI, 5-isoprostane F2alpha-VI, (8β)-5,9α,11α-trihydroxy-prosta-6E,14Z-dien-1-oic acid; 17(S)-HDoHE, 17S-hydroxy-4Z,7Z,10Z,13Z,15E,19Z-docosahexaenoic acid; LXA_5_, 5S,6R,15S-trihydroxy-7E,9E,11Z,13E,17Z-eicosapentaenoic acid; 15-HETE, 15-hydroxy-5,8,11,13-eicosatetraenoic acid; 9-HODE, 9-hydroxylinoleic acid, 9-hydroxy-10,12-octadecadienoic acid; 11,12-DHET, 11,12-dihydroxyeicosatrienoic acid; PGE_1_, Prostaglandin E_1_; PGF_2α_, Prostaglandin F_2α_; 13-HODE, 13-hydroxylinoleic acid, 13-hydroxy-10,12-octadecadienoic acid; PGE_2_, Prostaglandin E_2_; 14,15-DHET, 14,15-dihydroxyeicosatrienoic acid; 17-keto_DPA, 7Z,10Z,13Z,15E,19Z-17-oxo-docosapentaenoic acid; 8-iso-PGE_2_, 8-isoprostaglandin E_2_, 9-oxo-11α,15S-dihydroxy-(8β)-prosta-5Z,13E-dien-1-oic acid; 12-HETE, 12-Hydroxy-5,8,10,14-eicosatetraenoic Acid; AA, acids arachidonic acid. **(B)** All RvE1 values in FF of enrolled 80 patients were shown with cutoff value line (green dotted line). Data are presented as mean ± SD. Group A: blastocysts of high quality (blastocyst score ≥ B3BC and B3CB, *N* = 36); Group B: blastocysts of low quality (blastocyst score < B3BC and B3CB, *N* =44). **(C)** Receiver operating characteristic (ROC) curve analysis was used to determine the predictive power and cutoff value of RvE1 in FF of oocytes able or unable to form high-quality blastocysts.

A total of 80 patients underwent IVF progress were involved in the study on the correlation between metabolites and clinical outcomes. Subjects in group A (blastocysts scored ≥ B3BC and B3CB according to Gardner's blastocyst scoring system, *N* = 36) and group B (blastocysts scored < B3BC and B3CB, *N* = 44) showed similar levels in basic clinical characteristics, including age, body mass index (BMI) and baselines of estradiol (E2), progesterone (P4), FSH, and luteinizing hormone (LH). There was no significant difference between the two groups in terms of oocyte scores and 2-pronuclei counts while in Peter's score on Day 3, embryos from two groups differed (Table 1). LC/ESI-MS/MS was performed to quantify lipid metabolites including AA and other PUFAs in FF, revealing that group A contains a higher level of RvE1 (32.12 ± 3.84 pg/ml) than group B (18.07 ± 2.36 pg/ml, Figures 1A,B) with *P*-value = 0.0018 but no significant difference was found in other 36 metabolites (*P* >.05, Figure 1A). The results of receiver operating characteristic curve (ROC) analysis showed specificity of 97.2% and sensitivity of 25.0% (Figure 1C) with the optimal cutoff value of RvE1 (8.96 pg/ml) in FF from both groups determined by the largest positive likelihood ratio (8.99; value of sensitivity divided by 1 - specificity; Figures 1B,C). Area under ROC curve (AUC) was 0.75 (95%CI: 0.64–0.86, *P* < 0.00012, Figure 1C).

### RvE1 Receptor Is Expressed on CCs Rather Than on Oocytes

**Figure 2:**
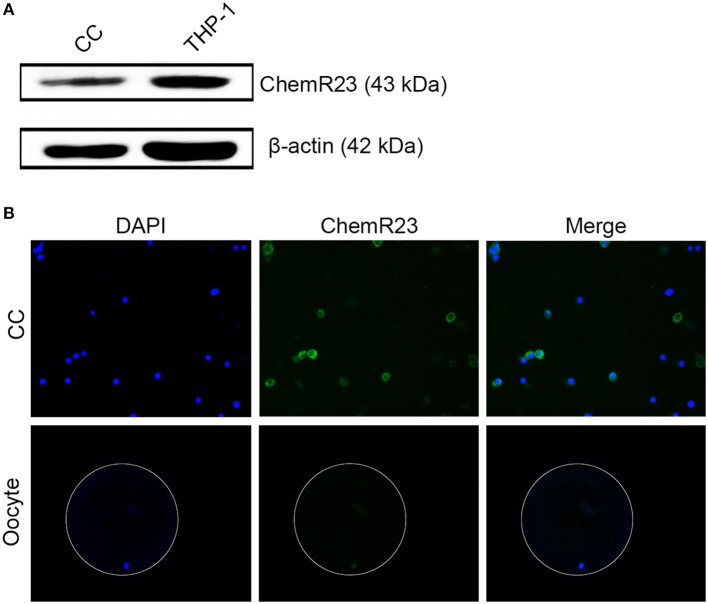
Identification of RvE1's receptor on cumulus cells (CCs) and oocytes. **(A)** Detection of ChemR23 expression on CCs by western blot. THP-1 as a positive control; β-actin as the internal reference. Three independent biological replicates. **(B)** The presence of ChemR23 (green) on CCs and oocyte was analyzed by fluorescence microscope. The nuclei of CCs and oocyte were stained with DAPI (blue). The outside silhouette of oocyte was presented by a white ring manually. Three independent biological replicates.

Immunoblotting and fluorescence microscopy displayed the positive expression of ChemR23 on CCs which was one of the most important receptors of RvE1 in humans (Figures 2A,B, Supplementary Figures 1A,B). Whereas, immunofluorescence showed that the receptor was expressed on oocytes (Figure 2B).

### RvE1 Increases the Viability and Stimulates the Growth of CCs

**Figure 3:**
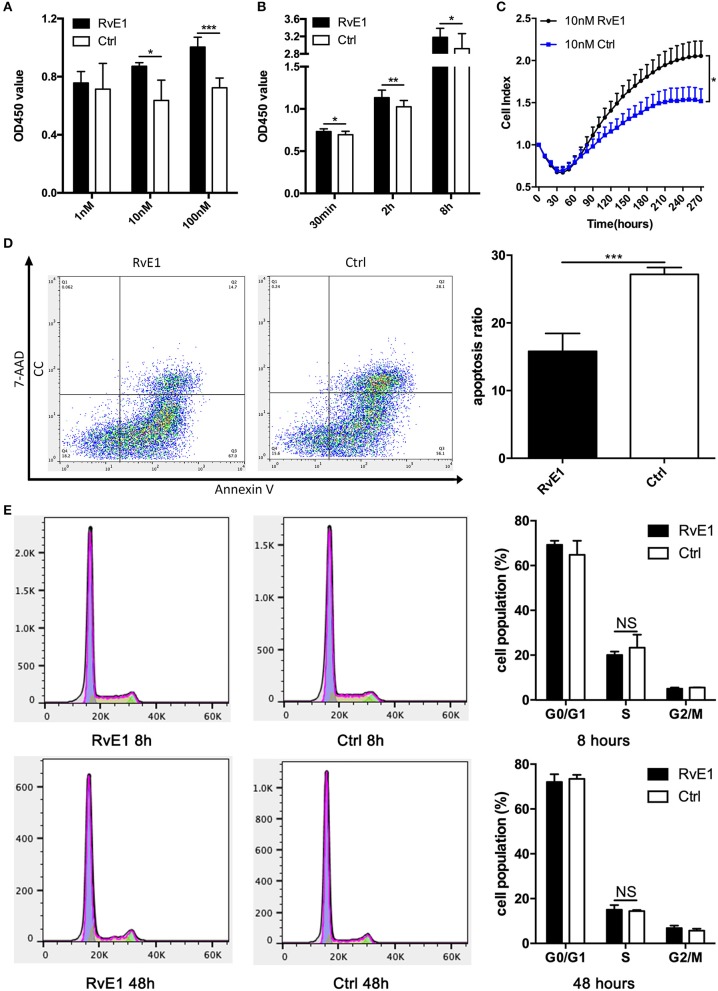
The effects of RvE1 on cumulus cells (CCs). **(A,B)** CCs' viability was measured by CCK-8 after treatment with 1, 10, and 100 nM RvE1 for 30 min **(A)** and 10 nM RvE1 for 0.5, 2, and 8 h **(B)**. Equal amounts of RvE1 solvent, anhydrous ethanol, as a negative control. Data are presented as mean ± SD; *p < 0.05; **p < 0.01; ***p < 0.001, multiple unpaired t-test with two tails using Holm-Sidak correction, three **(A)** and twelve **(B)** independent biological replicates. **(C)** Cell proliferation of 10 nM RvE1 treated CCs was detected by Label-free Real-time Cellular Analysis (RTCA). Equal amounts of anhydrous ethanol as negative control. Data are presented as mean ± SEM; *p < 0.05, multiple unpaired t-tests with two tails using Holm-Sidak correction, eight independent biological replicates. **(D)** Apoptosis of CCs treated with 10 nM RvE1 for 8 h. Equal amounts of anhydrous ethanol as a negative control. Data are presented as mean ± SD; ***p < 0.001; *t*-tests with two tails, five independent biological replicates. **(E)** The cell cycle of CCs with a treatment of 10 nM RvE1 for 8 and 48 h. Equal amounts of anhydrous ethanol as a negative control. Data are presented as mean ± SD; NS, *p* > 0.05, *t*-test with two tails, three independent biological replicates.

After treatment with 10 and 100 nM RvE1 for 30 min, the viability of CCs remarkably was increased in comparison with both negative control and lower dose group (Figure 3A). In a long-term analysis, the viability of CCs showed a significant increase in 10 nM RvE1 treated group compared with the solvent group at the time point of 0.5, 2, and 8 h (*P* = 0.043, 0.0038, and 0.036, respectively, Figure 3B). Label-free Real-time Cellular Analysis (RTCA) revealed that cell proliferation of CCs surged after treatment with 10 and 100 nM RvE1 (Figure 3C, Supplementary Figure 2A). However, there was no significant difference in cell proliferation between the CCs pretreated with 0.1 nM RvE1 and corresponding solvent control (Supplementary Figure 2B). As for cell cycle and cell apoptosis, RvE1 reduced the percentage of apoptotic cells (*p* = 0.00040, Figure 3D) but did not increase the proportion of S phase cells of CCs with *P*-values of 0.41 and 0.63 at 8 and 48 h after treatment (Figure 3E) in contrast with the control group containing equal amount of RvE1 solvent, anhydrous ethanol.

### Sources of RvE1 in FF

**Figure 4:**
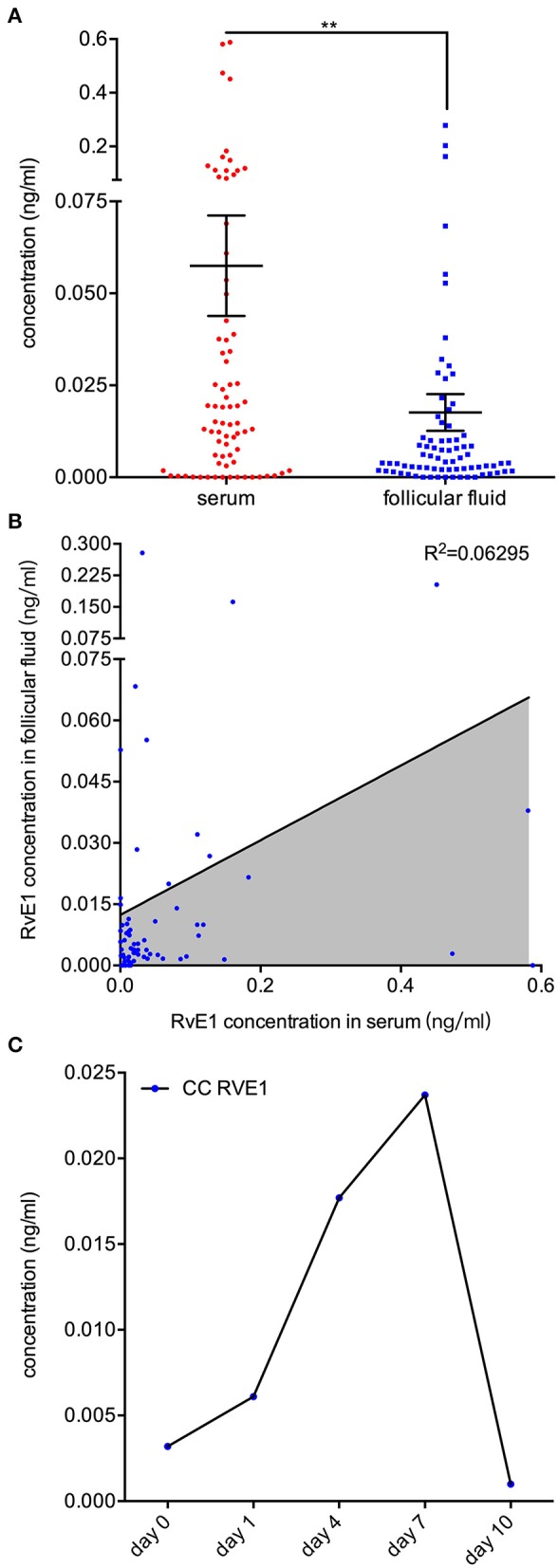
Sources of RvE1 in follicular fluid including both serum exudate and cumulus cells' (CCs) secretion. **(A)** RvE1 levels in follicular fluid (FF) and serum of 76 patients were analyzed by liquid chromatography-electrospray ionization tandem mass spectrometry (LC/ESI-MS/MS). Data are presented as mean ± SEM; **p < 0.01, paired t-test with two tails, 76 independent biological replicates. **(B)** Linear regression analysis was used to determine the relativity of the RvE1 level between FF and serum. The coefficient of determination was presented as R^2^, 76 independent biological replicates. **(C)** The level of RvE1 in conditioned medium of pooled CCs from 19 patients was analyzed by LC/ESI-MS/MS after being cultured for 0, 1, 4, 7, and 10 days.

**Figure 5:**
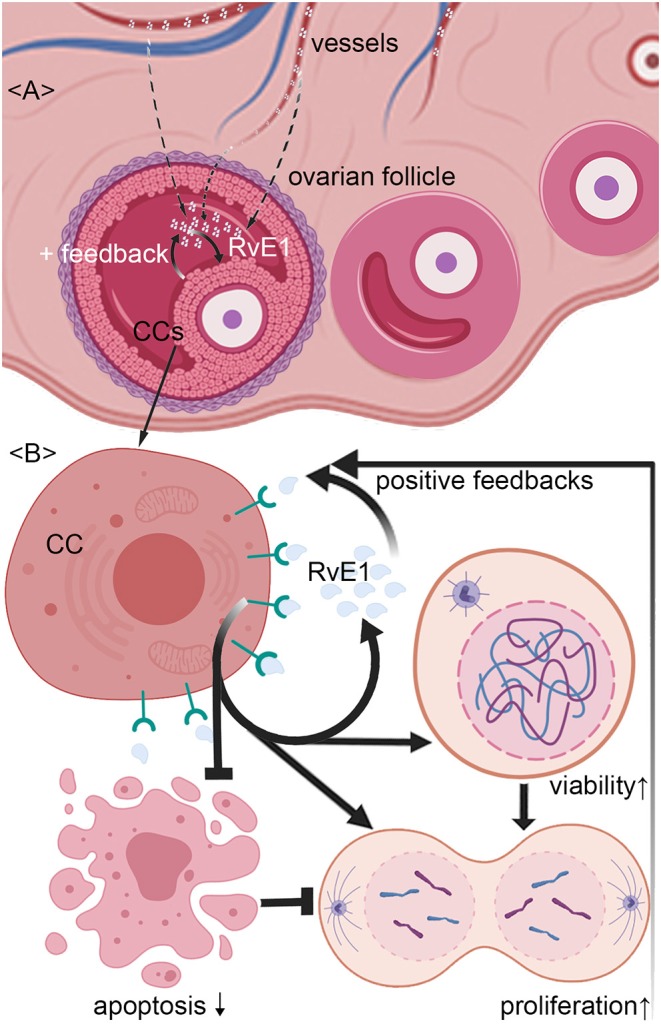
Schematic representation of mechanism by which RvE1 from serum and cumulus cells (CCs) contributes to a higher level of follicular fluid (FF) RvE1, improving oocyte quality through activating CCs' viability and promoting cell growth by attenuating apoptosis. The level of RvE1 in FF is elevated by both exudations of peripheral blood and secretion of CCs **(A)**. After binding to ChemR23, RvE1 enhances CCs' viability and promote their growth by inhibiting cell apoptosis **(B)**, which in turn promotes the production of RvE1 by CCs as positive feedback **(A,B)**.

To determine its sources *in vivo*, we examined the RvE1 level in both FF and serum collected from 76 women without metabolic disorders. Patients were aged from 24 to 43 years old with an average BMI of 21.0 (SD 2.6, range 16.0–31.6). The mean level of baseline hormones was 46.9 (SD 27.7, range 13.7–155.4) in E2, 1.8 (SD 2.5, range 0.6–18.6) in P4, 8.0 (SD 2.5, range 0.3–14.9) in FSH and 2.5 (SD 1.8, range 0.4–9.1) in LH. The mean numbers of retrieved oocytes and mature MII oocytes were 12.5 (SD 6.5, range 1–32) and 10.7 (SD 6.0, range 1–28), respectively. After *in vitro* fertilization, 9.0 ± 5.4 2-pronuclei oocytes were obtained and 5.0 ± 3.7 embryos of top quality were formed (Supplementary Table 1). The content of RvE1 was found to be higher in serum (57.49 ± 13.65 pg/ml) than in corresponding FF (17.62 ± 4.97 pg/ml, *P* < 0.0037, Figure 4A). Furthermore, the level of RvE1 in FF was partially linked to that in serum (*P* = 0.029, R^2^ = 0.063, Figure 4B). The culture medium of pooled CCs showed a rising level of RvE1 during the period of the first 7 days, whereas a dramatic decrease was observed on the day of 10 with masses of drifted CCs visually confirmed by microscopy (Figure 4C). The mechanism of RvE1 on CCs and oocytes was illustrated by a schematic representation (Figure 5).

## Discussion

In this study, we revealed the positive association between RvE1 and oocyte quality, suggesting RvE1 as a candidate for FF biomarkers to exclude inferior oocytes. It was the first time that the role of RvE1 in reproduction, more specifically, improving oocyte quality, was discovered and its cellular mechanisms were studied.

The prediction of developmental competence by biomarkers is considered to be of great importance to IVF outcome improvement (36). In contrast to assessments directly performed on oocytes or embryos (37, 38), FF, as the microenvironment where oogenesis and oocyte maturation take place, provides a noninvasive approach to estimate oocyte quality at a relatively early stage. And the efficacy of biomarkers found in FF has been confirmed in previous researches (39). In a retrospective cohort study, decorin in FF (F-DCN) was measured quantitively and showed a significantly lower content in FF of fertilized oocytes than that in unfertilized ones, which suggested the possibility and practicability of FF components as potential biomarkers to predict the quality of oocytes collected from corresponding follicles (40). Nevertheless, limited studies have focused on the predicting role of FF lipid metabolites, neither have their cellular mechanisms been illustrated (41). Actually, lipid metabolism which includes the processes of lipogenesis and lipolysis serves as an essential energy source for both oocyte maturation and subsequent embryonic development (42). Hence, the level of lipid metabolites may provide valuable insight into oocyte quality, facilitating a better understanding of the oocyte growth.

To screen out FF lipids that were indicative of oocyte competence, we retrieved the first follicles from both sides of ovaries in IVF patients and collected FF carefully and individually without visual blood contamination. Eighty samples of FF were detected for lipid metabolomic profiles by LC/ESI-MS/MS, an ideal technology developed to analyze metabolites in body fluid (43). The development of corresponding oocytes was observed constantly and individually. For the first time, RvE1 was screened out among 36 FF lipid metabolites and showed a significantly higher level in subjects attaining better blastocysts than those resulted in no blastomere formation or ended in failure to produce high-quality ones. The indicative ability of RvE1 was confirmed by ROC analysis with high specificity (97.22%) to exclude oocytes of unpreferable quality, allowing superior ones to proceeded furtherly and thus elevating the probability of IVF success.

CCs, a specific group of granulosa cells that are nearest to the oocyte, function as a natural barrier to regulate the exchange of substances between ovum and the outside of the follicle (44). In particular, CCs undergo a series of physiological activities after receiving exogenous stimulations and then transfer information to the enclosed oocyte through gap junctions such as Cx37 (45, 46). In order to find out whether RvE1 affected oocytes in a direct or indirect way, both immunoblotting and immunofluorescence were conducted. According to the results, ChemR23, the major receptors of RvE1, was confirmed to be absent on oocytes. It was rational to speculate that in the specific environment of FF, RvE1 might influence oocyte quality by regulating the physiology of contiguous cells, for instance, CCs, instead of function directly on oocytes. This presumption was also supported by the results of western blot and fluorescence microscopy that positive expression of ChemR23 was found on CCs. Phenotype tests were performed to explore the cellular mechanisms by which RvE1 regulated CCs' bioactivity. RvE1 was found to significantly elevate the viability of CCs as well as cell proliferation in a dose-dependent manner, suggesting that a certain level of RvE1 in FF facilitated oocyte development. With the aid of flow cytometry, the stimulatory effect of RvE1 on the growth of CCs was furtherly verified to exert through apoptosis inhibition rather than cell cycle promotion. Collectively, our results support the assumption that RvE1 influences oocytes by adjusting the biological behavior of CCs, which are in line with previous reports that oocyte competence was linked to the quantity and quality of CCs.

Physically, the content of lipid metabolites in FF fluctuates in accordance with the whole system level since FF is indeed an exudate of peripheral blood (47). However, several studies maintained the opposite notion (48). In order to find the source of RvE1 in FF, we collected samples of FF and serum from the same patient on the day of oocyte retrieval and detected RvE1's concentration by LC/ESI-MS/MS. Our findings demonstrated a positive association between FF RvE1 and serum RvE1 levels. Meanwhile, the opposite was also observed in particular subjects showing that FF RvE1 remained at a low level despite its significant-high content in serum. Considering the correlation coefficient R^2^ which was <0.07, it might be reasonable to conclude that the content of RvE1 in FF was but was not only influenced by peripheral blood circulation. To identify other origins of FF RvE1, CCs denuded from COCs of 19 patients were cultured as commixture *in vitro*. Regarding the high degradability of RvE1 in aqueous solution, the culture medium of CCs tended to provide “real-time” insight into the current level of FF RvE1 and thus reflect the present secretion of RvE1 by CCs. LC/ESI-MS/MS of culture medium represented that CCs considerably secreted RvE1 and this particular local production was found to be closely linked to the biological state of CCs since the secretion dropped sharply at the end of the culture period when CCs detached from the dish surface and floated. Taken together, these findings indicate that RvE1 was partly originated from circulation blood and partially produced by autocrine of CCs, which may promote the viability and cell proliferation of CCs by a mechanism of positive feedback.

In the present study, we successfully screened out a lipid biomarker, namely, RvE1, for oocyte quality assessment, which in turn added to the proof of FF metabolomics as a practical predictor of oocyte developmental competence. For the first time, the origin of RvE1in FF and its mediating mechanism in oocytes were explored, expanding the knowledge of FF metabolomics and deepening the understanding of the cross-talk between CCs and oocytes. The major merit of our findings lies in the possible assistance in discriminating superior oocytes from inferior ones at an early stage of IVF, which allows the establishment of a refined assessment system for predicting clinical outcomes of a single oocyte as well as the exclusion of unpromising oocytes. Consequently, subsequent culturing work in the laboratory may be focused on valuable eggs that are more probable to fertilize and develop into embryos with preferable viability. Only in this way can we improve IVF outcomes and save manpower and funding resources at the same time.

Despite significant findings, the current study has several limitations. In the part of clinical data collection, although we substituted end-point index of pregnancy and live birth by scores of oocytes and blastocysts in the consideration of multiple confounding factors during the period from oocyte maturation to delivery other than oocyte quality, it may result in a potential limitation of our study as an application basis. Additionally, the limited sample size might restrict statistic power. In terms of the basic experiment, there is a deficiency *in vivo* evidence that demonstrates the function of RvE1 of improving oocyte quality. Besides, the cell signaling pathway activated by RvE1 after combining to ChemR23 is yet to been discovered. To fulfill these research gaps, we will enlarge the scale of sample collection in the future study and confirm the efficacy of RvE1 by establishing animal models.

In brief, our current work illustrated that RvE1, derived from both the secretion of CCs and body circulation, positively related to oocyte competence and may improve oocyte quality by improving viability and inhibiting apoptosis of surrounding CCs. Therefore, RvE1 may be adopted as a potential exclusive biomarker for oocyte selection and serve as a promising intervention to improve the IVF success rate after being thoroughly investigated and rigorously testified in the future.

## Data Availability Statement

The datasets generated for this study can be found in MetaboLights, www.ebi.ac.uk/metabolights/MTBLS1488.

## Ethics Statement

The studies involving human participants were reviewed and approved by Shanghai Ji Ai Genetics & IVF Institute Ethics Committee. The patients/participants provided their written informed consent to participate in this study.

## Author Contributions

YZ: conception and design, experiments conduction, manuscript drafting, and data acquisition, analysis, and interpretation. ZZ: conception and design, experiments conduction, manuscript drafting, and data analysis and interpretation. HL: data acquisition, analysis, and interpretation. MZ: experiments conduction, data analysis, and interpretation. XP: conception and design and data analysis and interpretation. AX: experiments conduction and manuscript revising. RQ: experiments conduction and data analysis and interpretation. WH: experiments conduction. JF and XS: conception and design, financial support, and final approval of manuscript.

### Conflict of Interest

The authors declare that the research was conducted in the absence of any commercial or financial relationships that could be construed as a potential conflict of interest.
