# Supplementary material for: Resolvin E1 in Follicular Fluid Acts as a Potential Biomarker and Improves Oocyte Developmental Competence by Optimizing Cumulus Cells
Source: Front Endocrinol (Lausanne). 2020 Apr 16;11:210. doi: 10.3389/fendo.2020.00210 (PMC7176900; doi:10.3389/fendo.2020.00210)
Supplement: Supplementary file 1 [file Data_Sheet_1.pdf]

# *Supplementary Material*

## 1 Supplementary Figures and Tables

### 1.1 Supplementary Figures

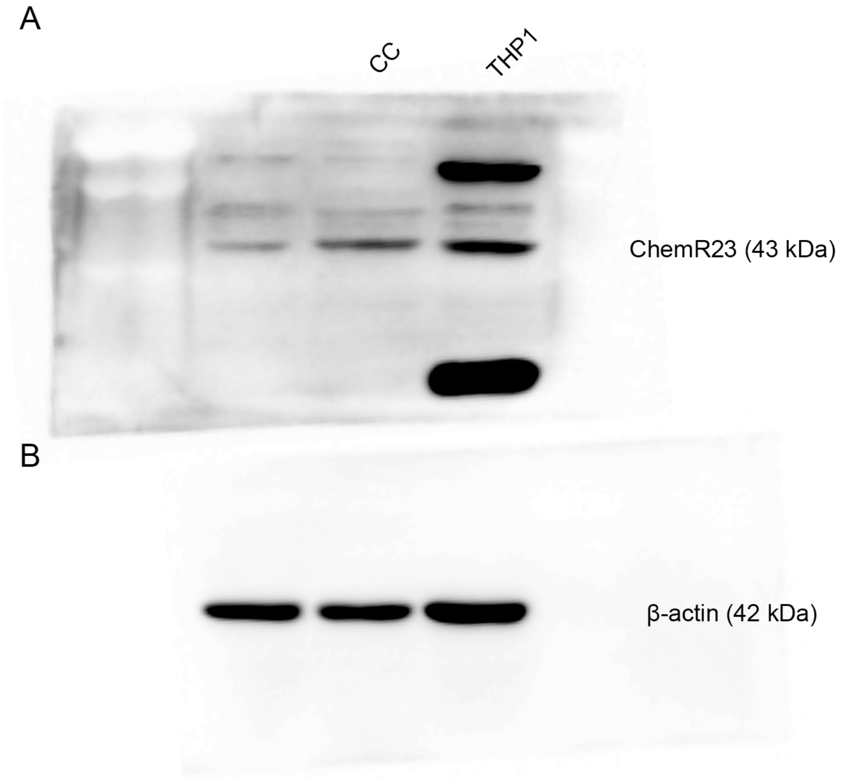


**Supplementary Figure 1.** Full gels of western blot. (A, B) Expression of ChemR23 and β-actin on cumulus cells (CCs).

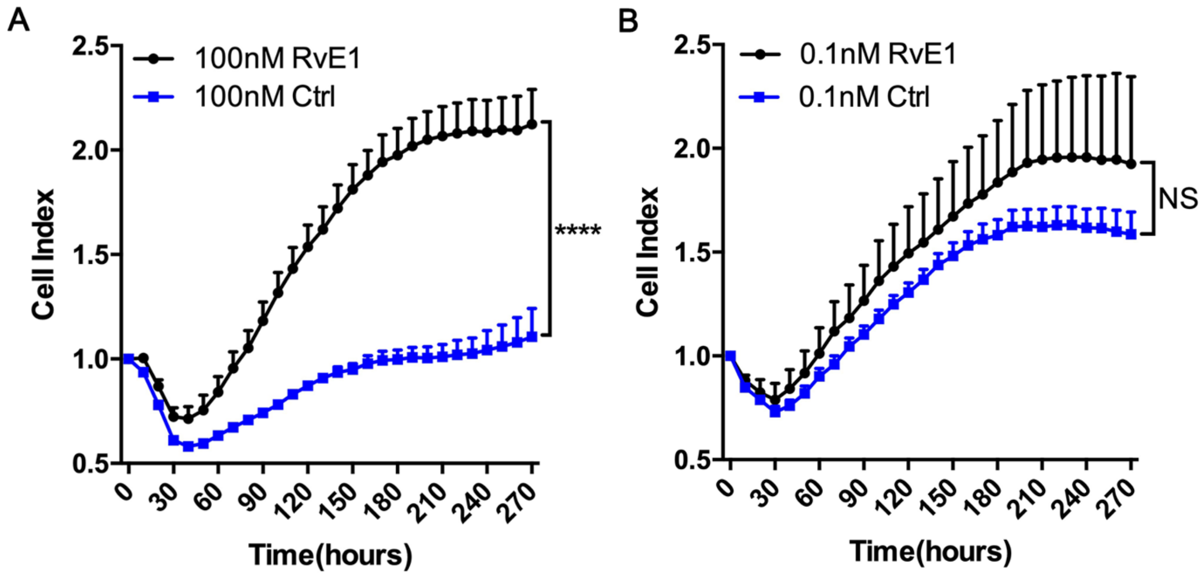


**Supplementary Figure 2.** The effect of RvE1 on the cell proliferation of cumulus cells (CCs).(A, B) Cell proliferation of CCs treated with 100 and 0.1 nM RvE1 was detected by Label-free Real-time Cellular Analysis (RTCA). Equal amounts of anhydrous ethanol as negative control. Data are presented as mean ± SEM; ****, $p < .0001$; NS, $p > .05$, multiple unpaired t-test with two tails using Holm-Sidak correction, eight independent biological replicates.
