# Supplementary material for: Resolvin E1 in Follicular Fluid Acts as a Potential Biomarker and Improves Oocyte Developmental Competence by Optimizing Cumulus Cells
Source: Front Endocrinol (Lausanne). 2020 Apr 16;11:210. doi: 10.3389/fendo.2020.00210 (PMC7176900; doi:10.3389/fendo.2020.00210)
Supplement: Supplementary file 2 [file Table_1.DOCX]

Supplementary Material

# Supplementary Figures and Tables

## Supplementary Table

**Supplemental Table 1:** Clinical characteristics of subjects undergoing targeted quantification of RvE1 in both serum and follicular fluid by liquid chromatography electrospray ionization tandem mass spectrometry (LC/ESI-MS/MS).

| Clinical parameter | Group C (N=76) |
| --- | --- |
| Age (years) | 31.9 ± 4.0 |
| BMI (kg/m2) | 21.0 ± 2.6 |
| E2 baseline (pg/ml) | 46.9 ± 27.7 |
| P4 baseline (ng/ml) | 1.8 ± 2.5 |
| FSH baseline (mIU/ml) | 8.0 ± 2.5 |
| LH baseline (mIU/ml) | 2.5 ± 1.8 |
| Number of retrieved oocytes | 12.5 ± 6.5 |
| Oocyte maturation |  |
| MII oocyte | 10.7 ± 6.0 |
| MI oocyte | 0.3 ± 0.8 |
| GV oocyte | 0.5 ± 1.2 |
| Fertilization |  |
| 2pn | 9.0 ± 5.4 |
| 1pn | 0.3 ± 0.5 |
| >2pn | 0.4 ± 0.8 |
| Embryo on Day3 |  |
| top quality embryos (grade 1-2) | 5.0 ± 3.7 |
| poor quality embryos (grade 3-4) | 3.9 ± 3.4 |

Data are mean ± SD. Group C: Newly enrolled IVF patients other than subjects in group A or B. Number of independent biological replicates was shown after N in the table. BMI, body mass index; E2, estradiol; P4, progesterone; LH, luteinizing hormone; FSH, follicle stimulating hormone; MII, oocyte at metaphase II; MI, oocyte at metaphase I; GV: germinal vesicle; pn :pronuclear; Day 3 embryo quality was graded according to modified Peter cleavage stage embryos scoring system with morphological criteria of number of blastomeres, blastomere regularity, granule occurrence and fragmentation rate.
